# Salvage surgery in recurrent sinonasal cancers: Proposal for a prognostic model based on clinicopathologic and treatment‐related parameters

**DOI:** 10.1002/hed.27102

**Published:** 2022-06-02

**Authors:** Davide Mattavelli, Michele Tomasoni, Marco Ferrari, Alessandra Compagnoni, Alberto Schreiber, Stefano Taboni, Vittorio Rampinelli, Elisa Marazzi, Elena Raffetti, Luca Oscar Redaelli de Zinis, Alberto Deganello, Roberto Maroldi, Paolo Bossi, Cesare Piazza, Piero Nicolai

**Affiliations:** ^1^ Unit of Otorhinolaryngology – Head and Neck Surgery ASST Spedali Civili of Brescia Brescia Italy; ^2^ Department of Medical and Surgical Specialties, Radiological Sciences, and Public Health University of Brescia Brescia Italy; ^3^ Section of Otorhinolaryngology – Head and Neck Surgery, Department of Neurosciences University of Padova – Azienda Ospedale‐Università di Padova Padova Italy; ^4^ Technology for Health (PhD Program), Department of Information Engineering University of Brescia Brescia Italy; ^5^ University Health Network (UHN) Guided Therapeutics (GTx) Program International Scholar, UHN Toronto Canada; ^6^ Epidemiology and Public Health Intervention Research Group (EPHIR), Department of Global Public Health Karolinska Institute Stockholm Sweden; ^7^ Unit of Radiology, Department of Medical and Surgical Specialties, Radiological Sciences, and Public Health University of Brescia Brescia Italy; ^8^ Unit of Medical Oncology, Department of Medical and Surgical Specialties, Radiological Sciences, and Public Health University of Brescia Brescia Italy

**Keywords:** prognostic score, recurrence, salvage surgery, sinonasal cancer, survival

## Abstract

**Background:**

Evidence on survival and major prognosticators after salvage surgery in recurrent sinonasal cancers (SNC) is limited.

**Methods:**

A retrospective, single‐center study of recurrent SNC treated with salvage surgery between 1997 and 2019 was conducted. Univariate and multivariable analyses were performed to define a prognostic score for overall survival (OS).

**Results:**

One hundred and eighteen patients were included. Recurrent SNC originated mostly in the naso‐ethmoidal box (67.8%) and were mainly epithelial (76.2%), high‐grade (49.2%), and locally advanced (rpT4, 60.1%) malignancies. Negative margins were achieved in 56.6% of cases. Two‐ and 5‐year OS were 71.7% and 56%, respectively. The prognostic model included treatment modality for primary tumor, histology, rpT class, margin status, perineural invasion, and adjuvant radiotherapy and stratified patients into three prognostic groups (5‐year OS: 84.4%, 44.9%, and 0%, respectively).

**Conclusions:**

Treatment of recurrent SNC can result in good long‐term survival estimates with limited morbidity. Our score can provide excellent prognostic stratification.

## 1 INTRODUCTION

Sinonasal cancers (SNC) represent a relevant treatment challenge for clinicians in view of their rarity, histologic heterogeneity, and critical site of origin. Survival is usually dismal, ranging between 50% and 70% at 5 years,^1^, ^2^, ^3^ and the recurrence rate is high, with about half of treated patients experiencing tumor relapse, mostly at the local site.^1^, ^4^

Local recurrences of SNC pose prominent challenges. In view of the low rate of distant spread (approximately in 10%–15% of cases),^2^, ^4^ achievement of local control is relevant to provide a higher chance of cure. On the other hand, the widespread use of upfront multimodal treatments may limit the array of adjuvant second‐line therapeutic options. Likewise, operating on heavy treated tissues is challenging from a technical standpoint and can be burdened by a high risk of complications.^1^

In this setting, the trade‐off threshold between aggressive and palliative treatments remains undetermined owing to the scarcity of published evidence. To date, only one study has specifically investigated prognostic indicators for salvage surgery in recurrent SNC.^5^ Thus, management of recurrent SNC is usually based upon the expertise of the multidisciplinary team. Moreover, the absence of a large amount of objective clinical data also hampers proper patient counseling and potentially leads to improper allocation of resources.

The present study is a retrospective analysis of a cohort of patients with recurrent SNC who underwent salvage surgery with or without adjuvant treatments at a single tertiary, academic, referral center. The aim is to define prognosticators for these patients and propose a prognostic score to predict survival.

## 2 MATERIALS AND METHODS

### 2.1 Study population

A retrospective analysis of consecutive patients affected by recurrent SNC treated from October 1997 to February 2019 was conducted at the Unit of Otorhinolaryngology – Head and Neck Surgery, ASST Spedali Civili, University of Brescia, Italy. Inclusion criteria were (a) first local recurrence of primary SNC (any malignant histology) after treatment with curative intent; (b) recurrence treated with curative intent through a surgery‐including protocol; (c) availability of survival outcomes with minimum follow‐up of 6 months for event‐free observations. Exclusion criteria included (a) persistent disease, defined as disease‐free interval (DFI) between primary and recurrent SNC <6 months, and (b) distant metastasis at recurrence.

The study was conducted in accordance with the Declaration of Helsinki and approved by the local ethics committee (NP3616).

### 2.2 Data collection and study definitions

Patients were selected from a prospectively accrued database and clinical‐pathological data (age, sex, origin of primary tumor, primary tumor treatment, DFI, surgery for recurrence, histology, grading, T classification, nodal status, tumor extension, orbit involvement, perineural invasion [PNI], lympho‐vascular invasion [LVI], surgical margins, adjuvant treatments) were retrieved by chart review. All tumors were reclassified according to the AJCC‐UICC TNM Staging System 8th Edition.^6^

Uninvolved surgical margins (R0) were defined as described in a previous publication from our group.^7^ Complications were categorized according to the Clavien–Dindo classification.^8^ In accordance with Kaplan et al.,^5^ we defined hospitalization ratio as the fraction of time spent in hospital after surgery out of overall survival (hospitalization time/total days alive following surgery).

### 2.3 Study objectives

The primary objective was the definition of recurrent SNC major prognosticators, and their combination in a prognostic score, having as main outcomes of interest overall (OS) and relapse‐free (RFS) survivals.

Secondary objectives were (a) definition of survival estimates for locoregional recurrence‐free survival (LRRFS) and distant recurrence‐free survival (DRFS) and (b) evaluation of safety of treatments.

### 2.4 Statistical analysis and development of a prognostic score

Characteristics of patients were expressed in terms of percentages, median, interquartile range (IQR), and range of values, as appropriate. Continuous variables were categorized according to their median value.

Univariate analyses were conducted using the Cox proportional hazard model and log‐rank test. Results were expressed in terms of hazard ratio (HR) and 5‐year OS estimates, respectively, with the relative 95% confidence intervals (CI), and graphically depicted by Kaplan–Meier curves.

A multivariable Cox proportional‐hazards model was conducted considering prognostically relevant clinical factors that may guide treatment in a recurrent setting. Variance inflation factor (vif) was estimated to exclude multicollinearity; vif <5 was considered as satisfactory. A prognostic formula, equivalent to the sum of the coefficients of risk for each independent prognosticator, was developed. The prognostic formula was applied in our cohort and a score was calculated for each patient.

To distinguish patients with poor, intermediate, and favorable prognosis, specific cut‐offs in the prognostic score were found by X‐tile software (3.6.1 – Yale University, New Haven, CT), according to the minimum *p* and the maximum *χ* ^2^ values. The X‐tile software generated randomized “training” and “validation” cohorts, which were normalized so that their base survival curves were similar. The “training” to “validation” cohort size ratio was 1:1. The minimum percentage of the total patient cohort for each subpopulation was set at 10%. Once the ideal cut‐off was automatically set in the “training” cohort, the software then internally validated it through its application in the “validation” cohort.

The so‐defined cutoffs were applied to the entire cohort. Survival curves with relative 95% CI and number of patients at risk by time according to the prognostic classification were plotted using the Kaplan–Meier method and compared with the log‐rank test. HR were retrieved with the Cox proportional hazard regression model.

Uni‐ and multivariate analyses for RFS were conducted using the same methodology. Statistical analysis was performed using R (version 4.0.4, R Foundation for Statistical Computing, Vienna, Austria) and *p‐*values <0.05 were considered statistically significant.

## 3 RESULTS

### 3.1 Clinical features of the series

During the study period, 543 patients with SNC were treated with curative intent at our institution; 118 (21.7%) met inclusion criteria. Eighty‐two (69.5%) patients were males and median age at recurrence was 66 years (IQR, 20.5; range, 20–88).

Tumor features are detailed in Table 1. Approximately two‐thirds (67.8%) of recurrent SNC primarily originated in the naso‐ethmoidal box, and 30.5% in the maxillary sinus. Median DFI between treatment for primary and recurrence was 18 months (IQR, 45.0; range, 6–338). Recurrent tumors were mostly epithelial malignancies (76.2%): intestinal‐type adenocarcinoma (ITAC, 29.7%), squamous cell carcinoma (SCC, 25.4%), and salivary gland‐type carcinoma (14.4%).

**TABLE 1:** Tumor features

Variable			*N* (%)
Primary tumor origin	Nasoethmoidal box		80 (67.8)
	Maxillary sinus		36 (30.5)
	Sphenoid sinus		2 (1.7)
Histology	Malignant epithelial tumors	Adenocarcinoma	36 (30.5)
		Intestinal‐type adenocarcinoma	35 (29.7)
		Nonintestinal‐type adenocarcinoma	1 (0.8)
		Squamous cell carcinoma	30 (25.4)
		Keratinizing	27 (22.9)
		Ex‐inverted papilloma	1 (0.8)
		Adenosquamous carcinoma	1 (0.8)
		Spindle cell carcinoma	1 (0.8)
		Salivary gland‐type carcinoma	17 (14.4)
		Adenoid cystic carcinoma	13 (11.0)
		Polymorphous adenocarcinoma	1 (0.8)
		Epithelial‐myoepithelial carcinoma	2 (1.7)
		Salivary duct carcinoma	1 (0.8)
		Sinonasal undifferentiated carcinoma	4 (3.4)
		Neuroendocrine tumors	3 (2.5)
		Atypical carcinoid	1 (0.8)
		Sinonasal neuroendocrine carcinoma	2 (1.7)
	Soft tissue tumors	Mesenchymal malignant tumors	12 (10.2)
		Chondrosarcoma	3 (2.5)
		Fibrosarcoma	4 (3.4)
		Leiomyosarcoma	1 (0.8)
		Malignant fibrous histiocytoma	1 (0.8)
		Malignant peripheral nerve sheath tumor	1 (0.8)
		Undifferentiated sarcoma	2 (1.7)
	Neuroectodermal tumors	Olfactory neuroblastoma	7 (5.9)
		Mucosal Melanoma	7 (5.9)
		Ewing sarcoma	1 (0.8)
	Germ cell tumors	Teratocarcinosarcoma	1 (0.8)
Tumor grade	Low grade		19 (16.1)
	Intermediate grade		41 (34.7)
	High grade		58 (49.2)
rpT classification (TNM 8th edition)	rpT1		16 (13.6)
	rpT2		16 (13.6)
	rpT3		15 (12.7)
	rpT4a		29 (24.5)
	rpT4b		42 (35.6)
rpN+	rcN0/rpN0		114 (96.6)
	rpN+		4 (3.4)
Anatomical structures involved by tumor	Nasoethmoidal box		84 (72.4)
	Maxillary sinus		45 (38.5)
	Frontal sinus		17 (14.5)
	Sphenoid sinus		26 (22.4)
	Anterior skull base bone		42 (35.9)
	Dura		17 (14.8)
	Brain		5 (4.3)
	Premaxillary soft tissues		18 (15.4)
	Superior alveolar process		13 (11.3)
	Hard palate		19 (16.2)
	Soft palate		4 (3.4)
	Nasopharynx		15 (12.8)
	Pterygopalatine Fossa		14 (12.2)
	Infratemporal Fossa		14 (12.0)
	Periorbit		19 (18.1)
	Extraconic fat		14 (13.2)
	Extrinsic ocular muscles		12 (11.0)
	Intraconic fat – orbital apex		8 (7.6)
	Lacrimal apparatus		15 (12.9)
PNI reported on final histology report	Pn0		98 (83.1)
	Pn1		20 (16.9)
LVI reported on final histology report	Lv0		102 (86.4)
	Lv1		16 (13.6)

Abbreviations: LVI, lymphovascular invasion; PNI, perineural invasion.

Recurrences were mostly classified as high‐grade (49.2%) and locally advanced (rpT4, 60.1%) lesions. The anterior skull base, dura, and brain were involved in 35.9%, 14.8%, and 4.3% of cases, respectively, while the nasopharynx, pterygopalatine (PPF), and infratemporal (ITF) fossae in 12.8%, 12.2%, and 12.0% of patients, respectively. Orbital involvement beyond the periorbita was recorded in 20 (16.9%) patients.

Details on treatments are summarized in Table 2. In most cases primary treatment was performed elsewhere (80.8%) and included chemo(C)‐radiation (RT) (61.5%), as either definitive (20.5%) or adjuvant (41.0%) therapy. For salvage surgery, a purely endoscopic procedure was performed in 52.2% of cases. Orbital exenteration was performed in 16.1% of patients. Median hospitalization time was 10 days (IQR, 8; range, 1–51). Negative surgical margins were achieved in 56.6% of cases. Adjuvant (C)RT was administered to 33 (30.0%) patients, and in 8 cases consisted of re‐irradiation (median DFI, 52.5 months). Treatment‐related mortality was 1.7%. The overall complication rate was 29.6%, with surgical or radiological intervention required in only 12.7% of cases (grade III complication according to the Clavien–Dindo classification).^8^

**TABLE 2:** Treatment‐related features

Variable		*N* (%)
Primary tumor treatment	Surgery	45 (38.5%)
	Surgery + adjuvant (Ch)RT	48 (41.0%)
	(Ch)RT	24 (20.5%)
Surgery for recurrence	Endoscopic resection	32 (27.4%)
	Endoscopic resection with transnasal craniectomy	29 (24.8%)
	Cranio‐endoscopic resection	16 (13.7%)
	Craniofacial resection	5 (4.3%)
	Medial maxillectomy	1 (0.8%)
	Inferior maxillectomy	3 (2.5%)
	Subtotal maxillectomy	3 (2.5%)
	Total maxillectomy	4 (3.4%)
	Total maxillectomy + orbital exenteration	19 (16.1%)
	Other	5 (4.3%)
Reconstruction of craniomaxillofacial defect (*N* = 28)	Free flap	18 (69.2%)
	Rectus abdominis	6 (23.1%)
	Anterolateral thigh	5 (19.2%)
	Latissimus dorsi	2 (7.7%)
	Radial forearm (+temporalis muscle)	1 (2) 3.8% (7.7%)
	Iliac crest	1 (3.8%)
	Scapular tip	1 (3.8%)
	Temporalis muscle	4 (15.5%)
	Obturator prosthesis only	3 (11.5%)
	No reconstruction	1 (3.8%)
	No data available	(2)
Extension of skull base surgery (*N* = 61)	Skull base bone	6 (10.3%)
	Dural resection	46 (79.3%)
	Brain parenchyma resection	6 (10.3%)
	No data available	(3)
Skull base reconstruction (*N* = 52)	Only graft (Ilio‐tibial tract)	29 (26) 67.4%
	Vascularized flap (w/o graft)	14 (32.6%)
	Septal mucosa	3 (7.0%)
	Pericranium	7 (16.3%)
	Temporoparietal fascial flap	3 (7.0%)
	Free flap	1 (2.3%)
	No data available	(9)
Resection of tumor with orbit at risk (*N* = 43)	Conservative (periorbita/extraconic fat resection)	24 (55.8%)
	Orbital exenteration	19 (44.2%)
Neck dissection	Performed	6 (5.1%)
	Non performed	112 (94.9%)
Surgical margins	R0 (resection with free margins)	64 (56.6%)
	R1 (microscopic positive margins)	36 (31.9%)
	R2 (macroscopic positive margins)	13 (11.5%)
Adjuvant (Ch)RT	Performed	33 (30.0%)
	Not performed	77 (70.0%)
Postoperative complications within 6 months	Death secondary to treatment (Grade V)	2 (1.7%)
	Grade III	15 (12.7%)
	Grade I–II	20 (16.9%)

Abbreviation: (Ch)RT, (chemo)radiotherapy.

### 3.2 Survival analysis

**FIGURE 1:**
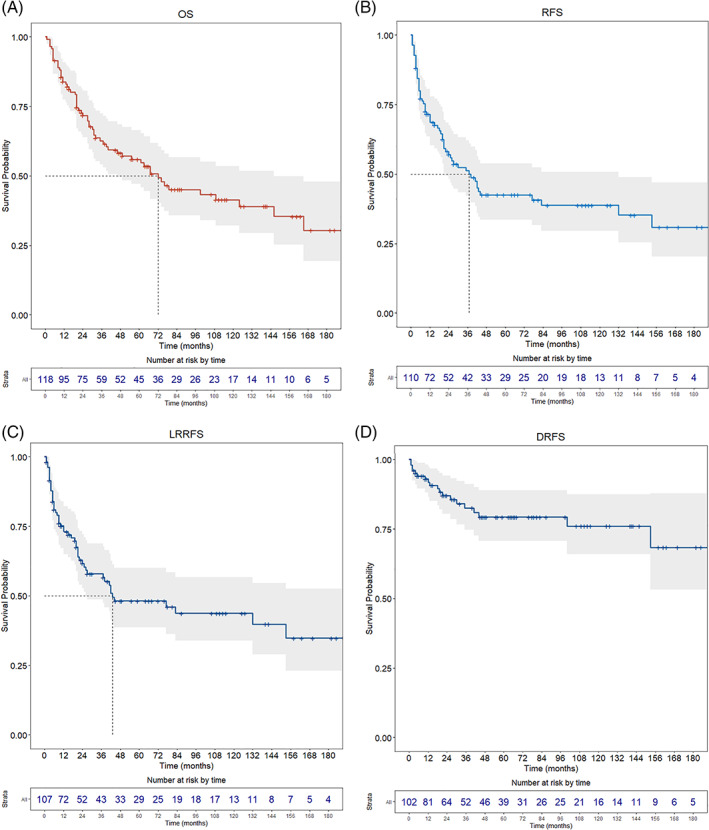
Kaplan–Meier survival curves depicting overall (OS), recurrence‐free (RFS), locoregional recurrence‐free (LRRFS), and distant recurrence‐free (DRFS) survivals, with relative 95% CI [Color figure can be viewed at wileyonlinelibrary.com]

Oncological outcomes and survival estimates are detailed in Table 3 and Figure 1.

**TABLE 3:** Oncological outcomes and survival estimates

		*N* (%)
Follow‐up		
Median follow‐up	36 (IQR, 67.5; range, 1–207)	
Survival		
Follow‐up status (at the end of the study)	Alive	57 (48.3)
	Free of disease	47 (39.8)
	With evidence of disease relapse	10 (8.5)
	Dead	61 (51.7)
	Dead of the disease	53 (44.9)
	Dead of other causes	8 (6.8)
Median survival time in dead patients ‐ months	23 (IQR, 40.5; range, 1–192)	
Overall survival (OS)		
1‐year OS (95% CI)	83.8% (77.4–90.7)	
2‐year OS (95% CI)	71.7% (63.9–80.6)	
5‐year OS (95% CI)	56.0% (47.1–66.5)	
10‐year OS (95% CI)	41.4% (32.0–53.6)	
Disease recurrence		
Further recurrence (any site)	Observed	61 (55.4)
	Not observed	49 (44.5)
Median DFI for further disease relapse (any site) – months	10 (IQR, 21.25; range, 1–153)	
Relapse‐free survival (RFS)		
1‐year RFS (95% CI)	68.6% (60.4–77.9)	
2‐year RFS (95% CI)	57.0% (48.2–67.5)	
5‐year RFS (95% CI)	42.5% (33.5–53.9)	
10‐year RFS (95% CI)	38.8% (29.6–50.8)	
Locoregional recurrence		
Further locoregional recurrence	Observed	52 (48.6)
	Not observed	55 (51.4)
Median DFI for 2nd locoregional recurrence – months	11 (IQR, 21.25; range, 1–153)	
Locoregional recurrence‐free survival (LRRFS)		
1‐year LRRFS (95% CI)	73.1% (65.0–82.1)	
2‐year LRRFS (95% CI)	61.6% (52.7–72.2)	
5‐year LRRFS (95% CI)	48.1% (38.6–60.0)	
10‐year LRRFS (95% CI)	43.7% (33.8–56.6)	
Distant recurrence		
Distant metastasis	Observed	19 (18.6)
	Non observed	83 (81.4)
Median DFI for distant metastasis – months	18 (IQR, 29.5; range, 1–153)	
Distant relapse‐free survival (DRFS)		
1‐year DRFS (95% CI)	91.8% (86.5–97.4)	
2‐year DRFS (95% CI)	87.0% (80.4–94.2)	
5‐year DRFS (95% CI)	79.3% (70.7–88.9)	
10‐year DRFS (95% CI)	76.0% (66.0–87.5)	

Abbreviations: CI, confidence intervals; IQR, interquartile range.

Median follow‐up was 36 months (IQR, 67.5; range, 1–207). At the last follow‐up, 57 (48.3%) patients were alive, and 47 (39.8%) were disease‐free. Sixty‐one (51.7%) were dead, mostly due to disease progression (53, 44.9%), showing a median survival time of 23 months (IQR, 40.5; range, 1–192). Further disease relapse (any site) was diagnosed in 61 (55.4%) patients, with a median DFI of 10 months (IQR, 21.25; range, 1–153). Locoregional recurrence was the most frequent site of tumor relapse (52 patients, 48.6%). Five‐year OS was 56.0%, while 5‐year RFS, LRRFS, and DRFS were 42.5%, 48.1%, and 79.3%, respectively.

The median hospitalization ratio among dead patients was 1.1%. At univariate analysis (Tables 4 and S1 and Figures S1 and S2, Supporting Information), maxillary localization, rpT classification, previous treatment including (C)RT (either exclusive or adjuvant), positive surgical margins, PNI, LVI, and high‐grade were strongly associated with reduced OS. Histology showed a significant impact on survival (Figure S1). Olfactory neuroblastoma (ONB), minor salivary gland carcinomas, and sinonasal undifferentiated carcinoma (SNUC) had the best survival estimates, whereas the poorest outcomes were recorded for recurrent sinonasal neuroendocrine carcinomas (SNEC), SCC, and mucosal melanoma (MM). Orbit involvement was associated with worsening OS, with a steady reduction in case of intraconal extension. Conversely, DFI between primary and salvage treatment had no prognostic impact (*p* = 0.203). The use of adjuvant RT (Figure S2) was associated with better survival outcomes (HR = 0.55, *p* = 0.074).

**TABLE 4:** Univariate analysis of variables affecting overall survival

Overall survival		Univariate analysis			
		Log‐rank test		Cox proportional hazard regression model	
Variable		5‐year OS (95% CI)	*p*‐value	HR (95% CI)	*p*‐value
Age at recurrence	<66 years old	58.7% (46.4–74.3)	0.308		
	≥66 years old	53.8% (42.1–68.9)		1.30 (0.78–2.17)	0.310
Sex	Female	60.5% (45.1–81.2)	0.442		
	Male	53.8% (43.5–66.6)		1.25 (0.71–2.21)	0.446
Origin of primary tumor	Nasoethmoidal box – sphenoid sinus	62.2% (51.9–74.5)	**0.024**		
	Maxillary sinus	41.6% (27.4–63.3)		1.83 (1.07–3.12)	**0.026**
Primary tumor treatment	Surgery	70.1% (57.2–85.9)	**0.004**		
	Surgery + adjuvant (Ch)RT	53.2% (39.7–71.1)		1.87 (1.01–3.48)	**0.046**
	Elective (Ch)RT	33.1% (17.9–61.2)		3.09 (1.55–6.15)	**0.001**
Disease‐free interval (DFI)	<18 months	44.2% (31.0–63.1)	0.077		
	≥18 months	65.9% (50.5–86.1)		0.56 (0.29–1.07)	0.079
Surgery for recurrence	Endoscopic resection	64.9% (48.6–86.6)	**0.002**		
	ERTC	81.2% (67.6–97.6)		0.90 (0.39–2.07)	0.803
	CER + CFR	46.4% (29.1–74.2)		1.86 (0.86–4.01)	0.115
	Maxillectomy	34.0% (17.2–67.4)		2.35 (1.08–5.11)	**0.031**
	Maxillectomy + CFR	30.3% (10.8–84.8)		4.15 (1.69–10.2)	**0.002**
Histology	Olfactory neuroblastoma	100%	**<0.001**		
	Squamous cell carcinoma	23.7% (11.6–48.7)		8.91 (2.04–38.92)	**0.004**
	Intestinal type carcinoma	64.3% (49.9–82.8)		2.73 (0.63–11.86)	0.181
	Mesenchymal malignancies	64.3% (41.2–100)		1.80 (0.33–9.85)	0.500
	Mucosal melanoma	33.3% (10.8–100)		7.46 (1.46–38.09)	**0.016**
	Neuroendocrine tumors	0%		61.2 (9.20–407.73)	**<0.001**
	Salivary gland‐type tumors	77.8% (54.9–100)		1.22 (0.20–7.43)	0.831
	Sinonasal undifferentiated carcinoma	75.0% (42.6–100)		1.36 (0.12–15.26)	0.801
Tumor grading	Low grade	82.1% (65.6–100)	**<0.001**		
	Intermediate grade	66.8% (53.0–84.3)		1.43 (0.56–3.63)	0.453
	High grade	40.4% (28.8–56.6)		3.58 (1.49–8.61)	**0.004**
rpT classification (TNM 8th edition)	rpT1	83.3% (64.7–100)	**<0.001**		
	rpT2	79.8% (61.7–100)		2.66 (0.66–10.75)	0.170
	rpT3	64.6% (43.9–95.1)		4.58 (1.25–16.84)	**0.021**
	rpT4a	60.9% (44.2–84.0)		3.67 (1.02–13.19)	**0.047**
	rpT4b	31.7% (19.8–50.8)		7.60 (2.29–25.18)	**<0.001**
Nodal status	rcN0/rpN0	72.5% (64.5–81.4)^a^	**0.028**		
	rpN+	50.0% (18.8–100)^a^		1.25 (1.07–11.35)	**0.038**
Tumor extension	Unilateral	56.3% (47.0–67.5)	0.973		
	Bilateral	48.6% (26.8–88.3)		0.98 (0.42–2.29)	0.968
Vectors of tumor extension			**0.004**		
Anterior (premaxillary soft tissues or nasal pyramid)	Absent	61.6% (52.2–72.6)			
	Present	22.7% (8.6–60.0)		2.51 (1.31–4.81)	**0.005**
Inferior (hard palate or superior alveolar process)	Absent	61.1% (51.6–72.4)	**0.014**		
	Present	31.5% (15.7–63.0)		2.10 (1.14–3.85)	**0.017**
Superior (skull base/dura/brain or sphenoid‐frontal)	Absent	65.1% (53.1–79.7)	**0.016**		
	Present	46.7% (34.9–62.4)		1.86 (1.11–3.09)	**0.017**
Posterior (soft palate or nasopharynx or PPF/ITF)	Absent	64.2% (54.5–75.7)	**<0.001**		
	Present	27.1% (13.6–54.1)		3.10 (1.79–5.37)	**<0.001**
Orbit involvement	Absent	67.4% (57.2–79.4)	**0.007**		
	Periorbit	40.0% (18.7–85.5)		1.68 (0.71–4.00)	0.241
	Extraconic fat	44.7% (20.7–96.7)		1.96 (0.75–5.13)	0.170
	Extrinsic muscles – Intraconic fat	11.4% (1.8–69.9)		3.63 (1.7–7.74)	**<0.001**
	Orbit apex	25.7% (5.2–100)		2.20 (0.90–5.65)	0.099
Perineural invasion	Pn0	61.5% (52.0–87.6)	**<0.001**		
	Pn1	28.2% (13.1–60.7)		2.75 (1.49–5.06)	**0.001**
Lymphovascular invasion	Lv0	59.7% (50.3–70.9)	0.092		
	Lv1	35.2% (17.6–70.0)		1.72 (0.91–3.25)	0.094
Surgical margins	Negative	67.4% (56.2–80.7)	**0.002**		
	Positive	37.7% (25.5–55.7)		2.22 (1.32–3.72)	**0.002**
Adjuvant (Chemo)radiation	Not performed	53.8% (43.2–67.0)	0.071		
	Performed	75.4% (61.0–93.2)		0.55 (0.28–1.06)	0.074

*Note*: The statistically significant *p*‐values (<0.05) are marked in bold.

Abbreviations: CER, cranio‐endoscopic resection; CFR, craniofacial resection; ERTC, endoscopic resection with transnasal craniectomy; ITF, infratemporal fossa; PPF, pterygopalatine fossa.

^a^ Two‐year OS.

### 3.3 Prognostic score

Multivariable analysis of the most relevant clinicopathological and treatment‐related features confirmed (C)RT for primary tumor, histology, rpT4b class, positive margins, PNI, and adjuvant RT after salvage surgery as independent prognosticators of OS (Table 5).

**TABLE 5:** Multivariable model based on the most relevant clinical‐pathological prognostic factors that may guide treatment in a salvage setting

Variable	Risk coefficient	HR (95% CI)	*p*‐value
Exclusive (Ch)RT for primary tumor	0.7877	2.20 (1.04–4.63)	0.038
Recurrent SCC	2.0894	8.08 (3.10–21.07)	<0.001
Recurrent ITAC	1.4322	4.19 (1.62–10.79)	0.003
Recurrent MM	3.0270	20.63 (5.08–83.88)	<0.001
Recurrent neuroendocrine tumors	2.5106	12.31 (2.71–55.82)	0.001
rpT4b	1.0343	2.81 (1.51–5.24)	<0.001
Positive surgical margins	1.3014	3.67 (1.81–7.44)	<0.001
Presence of perineural invasion	1.1330	3.10 (1.47–6.56)	0.003
No adjuvant RT after salvage surgery	1.0333	2.81 (1.32–5.99)	0.007

*Note*: Risk coefficients were used to determine the prognostic formula.

Abbreviations: (Ch)RT, (chemo)radiotherapy; ITAC, intestinal type adenocarcinoma; MM, mucosal melanoma; SCC, squamous cell carcinoma.

The risk coefficients of the abovementioned independent variables were used to develop our prognostic score, as follows:

**SCORE** = A*0.79 + B*2.09 + C*1.43 + D*3.03 + E*2.51 + F*1.03 + G*1.30 + H*1.13 + I*1.03.

**A**: Exclusive (C)RT for primary tumor (yes = 1; no = 0).

**B**: Recurrent SCC (yes = 1; no = 0).

**C**: Recurrent ITAC (yes = 1; no = 0).

**D**: Recurrent MM (yes = 1; no = 0).

**E**: Recurrent SNEC (yes = 1; no = 0).

**F**: rpT4b (yes = 1; no = 0).

**G**: Positive surgical margins (yes = 1; no = 0).

**H**: Presence of perineural invasion (yes = 1; no = 0).

**I**: No adjuvant RT after salvage surgery (yes = 1; no = 0).

**FIGURE 2:**
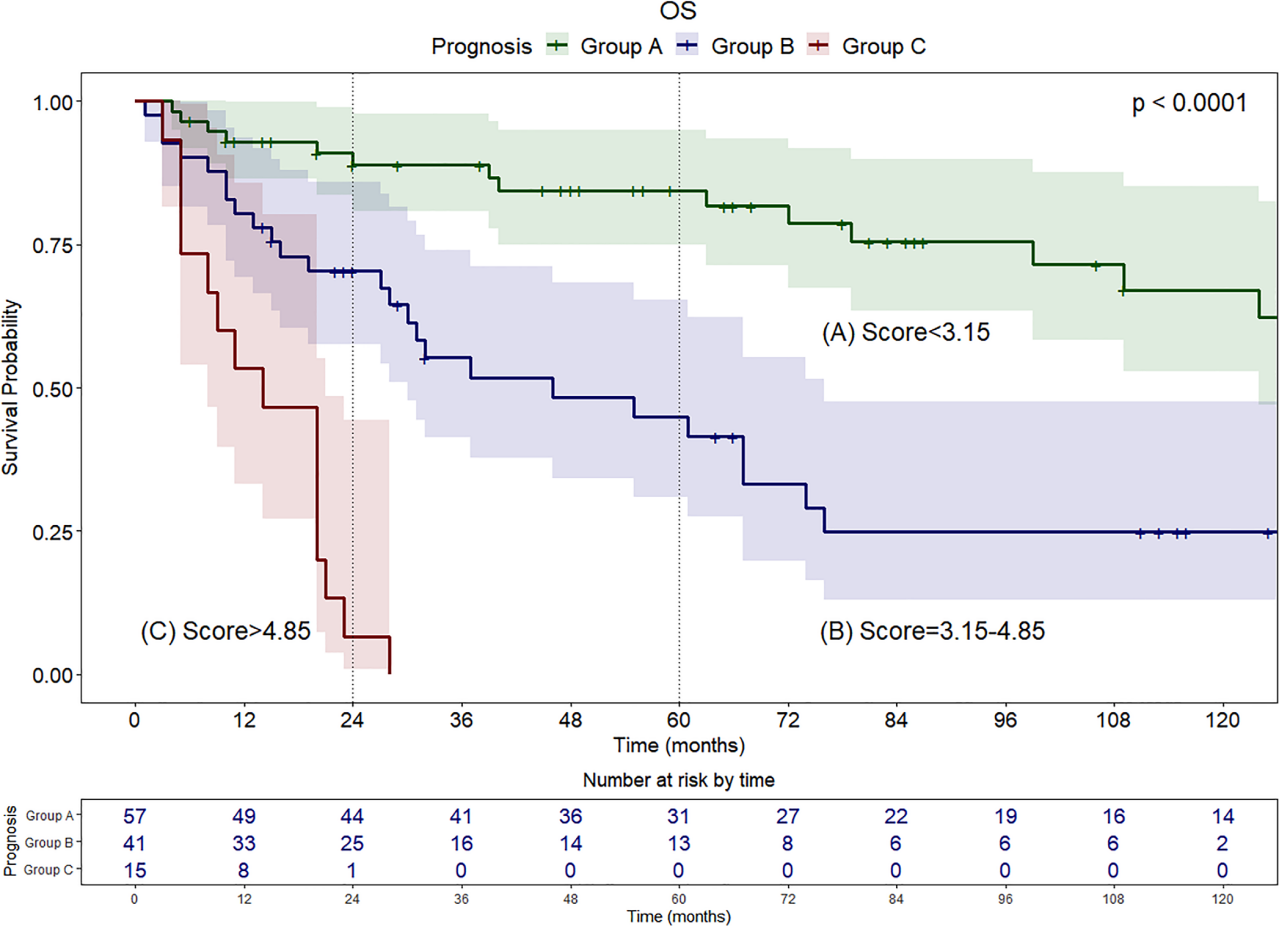
Kaplan–Meier survival curves and relative 95% CI (OS) according to prognostic classes (A: favorable, B: intermediate, C: poor) and relative score ranges found with the prognostic formula (cut‐offs found by X‐tile analysis) [Color figure can be viewed at wileyonlinelibrary.com]

According to the cut‐offs found with X‐tile analysis, patients were categorized into three groups: Group A (score <3.15) with favorable prognosis; Group B (score between 3.15 and 4.85) with intermediate prognosis; Group C (score >4.85) with poor prognosis (Figure 2). Median survival was 165, 46, and 14 months for Groups A, B, and C, respectively. Group A showed optimal survival even in the long‐term (2‐ and 5‐year OS of 88.8% [95% CI, 80.8–97.7] and 84.4% [95% CI, 74.9–95.0], respectively), whereas in Group B survival estimates decreased between the second (70.3% [95% CI, 57.5–86.0]) and fifth year (44.9% [95% CI, 30.8–65.3]). In Group C, only 1 patient among 15 (6.6%) survived 2 years after surgery, while none survived beyond 3 years. Compared to patients with good prognosis (Group A), a significant increase in mortality was observed for patients in Group B (HR, 3.71; 95% CI, 1.93–7.13; *p* < 0.001) and Group C (HR, 17.95; 95% CI, 7.77–41.46; *p* < 0.001).

### 3.4 Relapse‐free survival analysis

RFS largely paralleled OS. At univariate analysis (Table S2 and Figure S3), several variables were confirmed as negative prognosticators: maxillary location, type of previous treatment, surgical approach adopted, grading, rpT class, orbital involvement, and margin status. Histology reached a close‐to‐significant association with RFS (*p* = 0.088), while SCC, MM, SNEC, and SNUC showed the worst estimates. The multivariable model (Table S3) outlined the independent negative impact of SCC (HR, 2.40; *p* = 0.033), MM (HR, 3.22; *p* = 0.049), rpT4b class (HR, 1.88; *p* = 0.036), and positivity of surgical margins (HR, 2.43; *p* = 0.005) on tumor control. Adjuvant RT as part of salvage treatment showed a close‐to‐significant independent protective role (HR, 1.80; *p* = 0.085).

## 4 DISCUSSION

The present study demonstrates that aggressive treatment of recurrent SNCs through surgery‐including protocols can convey good survival estimates even in the long term. However, patient selection plays a pivotal role in optimizing outcomes, improving allocation of resources, and avoiding futile treatment‐related morbidity. Our score, based on the type of primary treatment, histology, rpT class, margin status, PNI, and adjuvant RT allows excellent discrimination of patients with recurrent SNC according to prognosis.

In the recurrent setting, achievement of local control is essential to provide the best chance of cure. In our series, the pattern of disease failure is dominated by further local relapses, representing the main cause of cancer‐related death. Survival estimates (5‐year OS of 56%, with a median survival of 2 years) are in line with the literature^5^, ^9^ and comparable, or slightly inferior, to those of primary SNC.^1^, ^3^ Accordingly, intensified treatment protocols including surgery are also recommended for recurrent SNCs and justified by the realistic possibility of achieving cure or, at least, prolonged survival. Orlandi et al. recently analyzed the impact of multimodal treatments in 69 stage III–IV epithelial SNCs.^10^ Among 44 patients who recurred locally, 19 were amenable to salvage surgery. Survival after treatment of tumor relapse was longer in patients who underwent surgery compared to responders to palliative chemotherapy.

A major open issue is the possibility to discriminate among patients who can benefit from intensified re‐treatments, and those who are likely to experience early failure and poor survival regardless of an aggressive therapeutic strategy. Kaplan et al. revised their series of 42 recurrent SNCs and proposed a therapeutic algorithm which included histology, site of recurrence, and tumor extension as key parameters.^5^ Briefly, they strongly supported surgery in case of low‐risk histology (ONB and adenoid cystic carcinoma), low‐grade tumor, and no extension to the orbit or skull base; on the other hand, they advised palliative care in case of high‐risk, high‐grade lesions with orbital or skull base invasion, evaluating curative treatments on a case‐by‐case basis if the recurrence originated in the naso‐ethmoidal complex.

The score presented herein includes a wider set of prognosticators and provides a quantitative estimate of survival probability for the individual patient. Group A included subjects with excellent survival estimates, which was maintained even in the long term. Group B included patients with intermediate prognosis: the treatment strategy may still be curative, but the risk of failure in the long term is greater. Finally, in Group C survival estimates were comparable to those of metastatic head and neck cancers. In these cases, morbidity of treatment should be cautiously weighed against the limited chance of survival. Evaluation of patient age, performance status, and his/her compliance/motivation should play a pivotal role within a thorough multidisciplinary discussion involving, as much as possible, the patient and his/her caregiver(s).

As for primary SNCs, even in the recurrent setting histology and tumor biology are critical and largely drive the prognosis despite any intensified treatment. In fact, in our series, aggressive histology (i.e., SCC, SNEC, and MM) and PNI were major negative prognosticators irrespective of T classification, margin status, and adjuvant treatments.

Surprisingly, DFI showed only a marginal influence on survival. Even in other smaller series, it did not reach statistical significance.^5^, ^11^ This could be related to the wide spectrum of sinonasal histologies with diverse biologic behavior, some of which display indolent growth and a tendency for delayed recurrence, while others are characterized by rapid and dismal progression.

Delivering adjuvant treatments was protective for further recurrence and improved survival. In addition to selecting radioresistant and dedifferentiated clones, primary CRT may also have a negative prognostic impact by limiting the use of adjuvant treatments in the recurrent setting, even when indicated. In fact, in our series, the use of adjuvant (C)RT after salvage surgery was relatively low (30%) compared to the high rate of negative major prognosticators (i.e., rpT4 [60.1%] and positive margins [43.4%]), and re‐irradiation was proposed to only eight patients. These findings should prompt clinicians to carefully evaluate the overall therapeutic margin before treating recurrent SNCs, and possibly consider re‐RT more favorably in highly selected cases. In a recent series, re‐RT with stereotactic technique or protons showed promising results with acceptable toxicity and definitively warrants further investigation.^11^, ^12^

Differently than expected, in our series the safety profile was deemed acceptable, with limited treatment‐related mortality, low complication rates, and short duration of hospitalization. Even in dead patients, hospitalization time was negligible (1.1%). Our findings are in line with those of Kaplan et al. which support a relatively low threshold to propose salvage surgery even in patients with comorbidities.^5^ Our study was not powered to analyze the long‐term effects on quality of life; this issue deserves focused investigations in future studies.

Lastly, some limitations of the study should be outlined. First, the retrospective design implies all the classical bias related to data retrieval and lack of control on treatment decisions, although this is minimized by the monocentric nature of the series. Second, there is a risk of overfitting of the model, and external validation with possible refinements in the variables considered or their relative weight is mandatory. Third, we could not include parameters related to the performance and nutritional status of patients (i.e., Karnofsky, preoperative albumin, etc.), as well as preoperative blood markers, because they could be retrieved only for a small proportion of the series. Finally, the score includes parameters that are available only at pathological report (rpT4b, margin status, and PNI), which prevents its use in the therapeutic decision‐making process. However, pT classification can be allegedly predicted through adequate preoperative imaging; PNI may sometimes be inferred from the pathologic evaluation at first diagnosis, and the probability to achieve free margins may be anticipated based on tumor extension and the surgeon's evaluation.

## 5 CONCLUSIONS

Intensified treatment of recurrent SNCs can lead to prolonged survival, with OS estimates that are close to those of primary SNCs. Patient selection plays a crucial role, and herein we propose a prognostic score that is able to stratify those with excellent, intermediate, or extremely poor prognosis. The overall safety profile is satisfactory. Retreatment protocols are recommended in recurrent SNCs and should be thoroughly discussed in a multidisciplinary setting.

## CONFLICT OF INTEREST

The authors declare that there is no conflict of interest that could be perceived as prejudicing the impartiality of the research reported.

## Supporting information


**Appendix S1** Supporting Information.

## Data Availability

The data that support the findings of this study are available from the corresponding author upon reasonable request.
