# Supplementary material for: Salvage surgery in recurrent sinonasal cancers: Proposal for a prognostic model based on clinicopathologic and treatment‐related parameters
Source: Head Neck. 2022 Jun 2;44(8):1857–70. doi: 10.1002/hed.27102 (PMC9539884; doi:10.1002/hed.27102)

**SUPPLEMENTARY TABLES**

**Table S1.** Prognosis of patients with critical tumor extension (overall survival).

| **Overall Survival** | | **Univariate analysis** | | | |
| --- | --- | --- | --- | --- | --- |
|  |  | **Log-rank test** | | **Cox proportional hazard regression model** | |
| **Tumor extension to:** | |  |  |  |  |
| **Anterior skull base bone - Dura** | Absent | 60.9% (49.8-74.4%) |  | REF |  |
|  | Present | 48.4% (35.0-66.8%) | **0.032** | 1.74 (1.04-2.89) | **0.034** |
| **Sphenoid or frontal sinus** | Absent | 62.7% (52.3-75.1%) |  | REF |  |
|  | Present | 41.3% (27.3-62.4%) | **0.038** | 1.73 (1.03-2.91) | **0.038** |
| **Nasopharynx** | Absent | 62.6% (53.2-73.6%) |  |  |  |
|  | Present | 15.6% (4.4-54.5%) | **<0.001** | 3.91 (2.03-7.51) | **<0.001** |

|  |
| --- |

**Table S2.** Univariate and multivariable analysis of variables affecting Relapse-Free Survival (RFS).
(Ch)RT – (Chemo)Radiation Therapy, CER – Cranioendoscopic resection, CFR – Craniofacial resection, ER – Endoscopic resection, ERTC – Endoscopic resection with transnasal craniectomy, ITAC – Intestinal type adenocarcinoma, ITF – infratemporal fossa, MM – Mucosal Melanoma, NE – Nasoethmoidal, ONB – Olfactory neuroblastoma, PPF – pterygopalatine fossa, SCC – Squamous cell carcinoma, SNUC – Sinonasal Undifferentiated carcinoma, uERTC – Unilateral Endoscopic resection with transnasal craniectomy; * referred to 1-y OS.

| **Relapse Free Survival (RFS)** | | **Univariate analysis** | | | |
| --- | --- | --- | --- | --- | --- |
|  |  | **Log-rank test** | | **Cox proportional hazard regression model** | |
| **Variable** | | **5-y RFS (95% CI)** | **p-value** | **HR (95% CI)** | **p-value** |
| **Age at recurrence** | <66-year-old | 45.2% (33.2-61.6%) | 0.452 | REF |  |
|  | ≥66-year-old | 39.3% (27.3-56.7%) |  | 0.82 (0.50-1.36) | 0.449 |
| **Gender** | Female | 53.3% (37.9-75.0%) | 0.457 | REF |  |
|  | Male | 38.1% (27.9-52.1%) |  | 1.24 (0.71-2.17) | 0.451 |
| **Origin of primary tumor** | NE box - Sphenoid sinus | 50.4% (39.5-64.2%) | **0.009** | REF |  |
|  | Maxillary sinus | 25.4% (13.6-47.3%) |  | 1.97 (1.17-3.32) | **0.011** |
| **Primary tumor treatment** | Surgery | 67.6% (54.0-84.8%) | **0.001** | REF |  |
|  | Surgery + adjuvant (Ch)RT | 22.6% (12.4-40.9%) |  | 2.96 (1.59-5.50) | **<0.001** |
|  | Exclusive (Ch)RT | 32.0% (15.9-64.1%) |  | 2.67 (1.59-5.50) | **<0.001** |
| **Disease Free Interval (DFI)** | <18 months | 45.4% (31.3-65.9%) | 0.616 | REF |  |
|  | ≥18 months | 34.7% (20.6-58.5%) |  | 1.17 (0.63-2.16) | 0.622 |
| **Surgery for recurrence** | ER | 59.8% (42.6-83.9%) | **0.004** | REF |  |
|  | u-ERTC + ERTC | 58.0% (41.7-80.6%) |  | 1.06 (0.48-2.37) | 0.883 |
|  | CER + CFR | 26.8% (12.7-56.5%) |  | 2.42 (1.12-5.25) | **0.025** |
|  | Maxillectomy | 17.4% (5.2-57.8%) |  | 2.71 (1.21-6.04) | **0.015** |
|  | Maxillectomy + CFR | Insufficient follow-up |  | 3.49 (1.41-8.62) | **0.007** |
| **Histology** | ONB | 71.4 (44.7-100%) | *0.088* | REF |  |
|  | SCC | 28.6% (13.6-60.1%) |  | 2.71 (0.90-8.14) | *0.075* |
|  | ITAC | 52.1% (36.7-74.1%) |  | 1.27 (0.42-3.82) | 0.676 |
|  | Mesenchymal malignancies | 63.6% (40.7-99.5%) |  | 0.84 (0.21-3.35) | 0.801 |
|  | MM | 22.2% (4.1-100%) |  | 2.08 (0.51-8.45) | 0.306 |
|  | Neuroendocrine tumors | 0% |  | 6.68 (1.17-38.14) | **0.033** |
|  | Salivary gland tumors | 35.9% (16.6-77.6%) |  | 1.62 (0.50-5.33) | 0.423 |
|  | SNUC | 25.0% (4.6-100%) |  | 2.25 (0.50-10.11) | 0.291 |
| **Tumor grading** | Low grade | 60.2% (41.0-88.3%) | **0.004** | REF |  |
|  | Intermediate grade | 53.8% (39.2-73.8%) |  | 1.28 (0.56-2.93) | 0.559 |
|  | High grade | 26.3% (15.5-44.6%) |  | 2.75 (1.26-6.01) | **0.014** |
| **pT Classification (TNM 8th edition)** | rpT1 | 67.4% (45.1-100%) | **0.025** | REF |  |
|  | rpT2 | 60.3% (40.0-90.9%) |  | 1.34 (0.42-4.25) | 0.617 |
|  | rpT3 | 34.4% (15.7-75.5%) |  | 2.17 (0.71-6.65) | 0.249 |
|  | rpT4a | 45.2% (27.8-73.5%) |  | 1.94 (0.70-5.38) | 0.129 |
|  | rpT4b | 23.7% (12.6-44.2%) |  | 3.50 (1.33-9.17) | **0.010** |
| **Nodal status** | pN0* | 69.6% (61.3-79.0%) | 0.128 | REF |  |
|  | pN+* | 33.3% (6.7-100%) |  | 2.94 (0.71-12.18) | 0.138 |
| **Tumor extension** | Unilateral | 42.5% (33.1-54.6%) | 0.784 | REF |  |
|  | Bilateral | 46.7% (23.3-93.6%) |  | 0.87 (0.35-2.18) | 0.771 |
| **Vectors of tumor extension** | |  |  |  |  |
| **Anterior** | Absent | 44.7% (35.1-57.0%) | 0.143 | REF |  |
| (premaxillary soft tissues or nasal pyramid) | Present | 27.7% (11.0-69.9%) |  | 1.70 (0.84-3.48) | 0.143 |
| **Inferior** | Absent | 46.1% (36.3-58.6%) | **0.040** | REF |  |
| (hard palate or superior alveolar process) | Present | 23.5% (9.2-60.2%) |  | 1.93 (1.02-3.66) | **0.042** |
| **Superior** | Absent | 51.9% (39.7-67.9%) | **0.038** | REF |  |
| (skull base/dura/brain or sphenoid-frontal) | Present | 31.6% (20.2-49.4%) |  | 1.70 (1.02-2.82) | **0.040** |
| **Posterior** | Absent | 51.4% (41.1-64.1%) | **<0.001** | REF |  |
| (soft palate or nasopharynx or PPF/ITF) | Present | 6.6% (1.0-42.6%) |  | 2.74 (1.57-4.79) | **<0.001** |
| **Orbit content** | Absent | 51.4% (40.3-65.4%) | **<0.001** | REF |  |
|  | Periorbit | 33.3% (13.2-84.0%) |  | 2.02 (0.89-4.56) | *0.091* |
|  | Extraconic fat | 46.7% (23.3-93.6%) |  | 1.57 (0.61-4.05) | 0.351 |
|  | Extrinsic muscles - Intraconic fat | Insufficient follow-up |  | 3.34 (1.58-7.06) | **0.002** |
|  | Orbit apex | 0% |  | 4.37 (1.69-11-33) | **0.002** |
| **Perineural invasion** | Pn0 | 44.4% (34.8-56.7%) | 0.356 | REF |  |
|  | Pn1 | 27.7% (9.5-80.6%) |  | 1.37 (0.69-2.73) | 0.364 |
| **Lymphovascular invasion** | Lv0 | 45.4% (35.8-57.7%) | 0.132 | REF |  |
|  | Lv1 | 21.4% (6.8-67.8%) |  | 1.67 (0.85-3.31) | 0.138 |
| **Surgical margins** | R0 (resection with free margins) | 52.6% (40.7-68.0%) | **0.005** | REF |  |
|  | R+ (positive surgical margins) | 28.3% (16.8-47.6%) |  | 2.05 (1.22-3.45) | **0.006** |
| **Adjuvant (Ch)RT** | Not performed | 39.1% (28.6-53.5%) | 0.225 | REF |  |
|  | Performed | 52.0% (36.4-74.3%) |  | 0.70 (0.39-1.24) | 0.224 |

**Table S3.** Multivariable model based on the most relevant clinical-pathological prognostic factors that may guide treatment in a salvage setting applied according to RFS.

| **Variable** | **Risk coefficient** | **HR (95%CI)** | **p-value** |
| --- | --- | --- | --- |
| Exclusive (Ch)RT for primary tumor | 0.2231 | 1.25 (0.58-2.68) | 0.566 |
| Recurrent SCC | 0.8745 | 2.40 (1.07-5.36) | **0.033** |
| Recurrent ITAC | 0.2144 | 1.24 (0.57-2.69) | 0.587 |
| Recurrent MM | 1.1683 | 3.22 (1.00-10.31) | **0.049** |
| Recurrent neuroendocrine tumors | 0.7500 | 2.12 (0.43-10-42) | 0.356 |
| rpT4b | 0.6339 | 1.88 (1.04-3.41) | **0.036** |
| Positive surgical margins | 0.8865 | 2.43 (1.31-4.51) | **0.005** |
| Presence of perineural invasion | 0.0603 | 1.06 (0.49-2.32) | 0.879 |
| No adjuvant RT after salvage surgery | 0.5902 | 1.80 (0.92-3.53) | *0.085* |

**SUPPLEMENTARY FIGURES**

**Figure S1.** Kaplan Meier survival curves depicting overall survival (OS) according to (a) most relevant histologies (“other histologies” includes ONB, salivary, mesenchymal tumors and SNUC); (b) rpT classification; (c) presence of perineural invasion (PNI); (d) tumor grading (low-G1, intermediate-G2, high-G3).

**
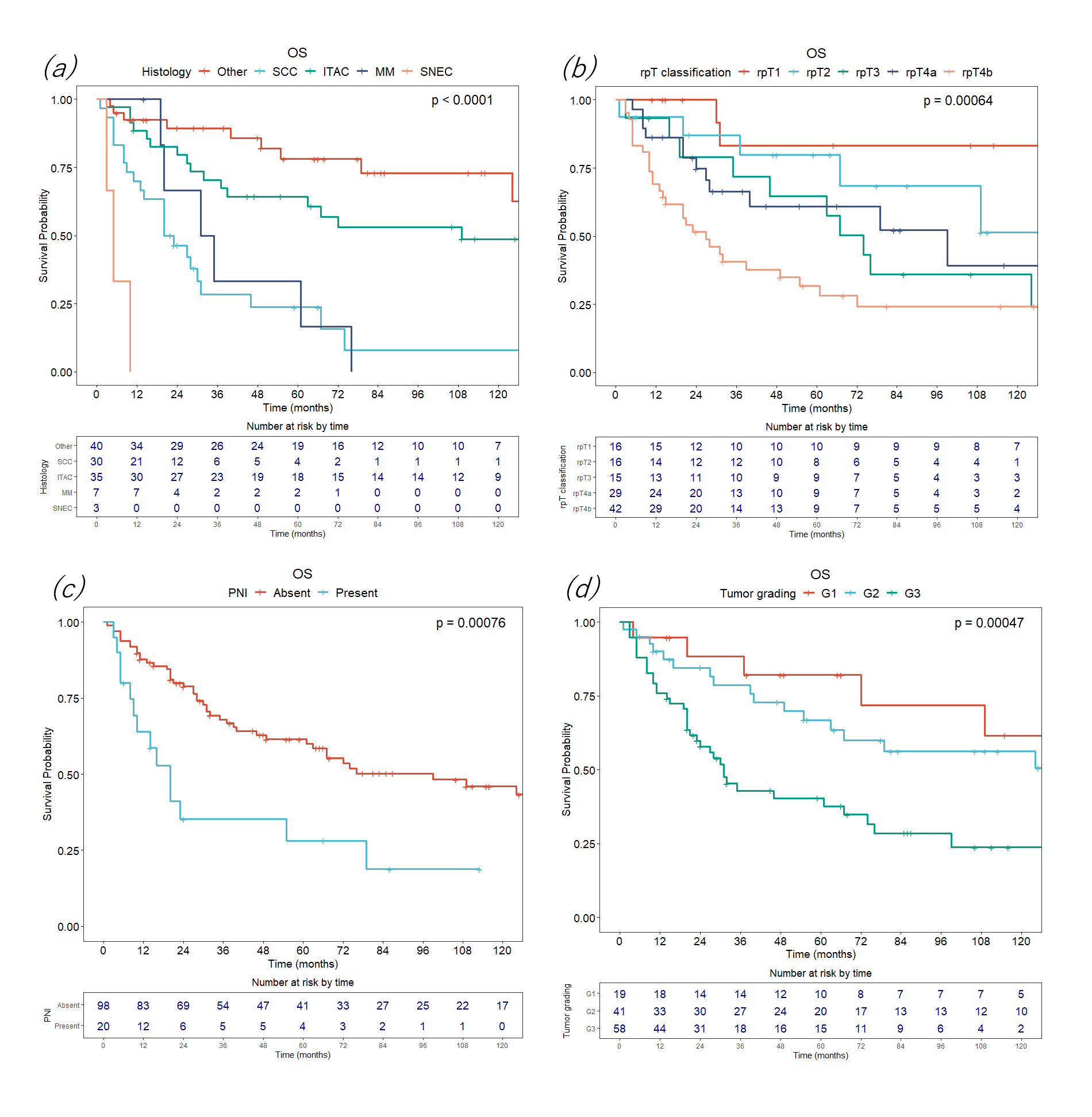
**

**Figure S2.** Kaplan-Meier survival curves (OS) according to previous treatment for primary tumor, margin status, and adoption of adjuvant RT after salvage surgery.

**
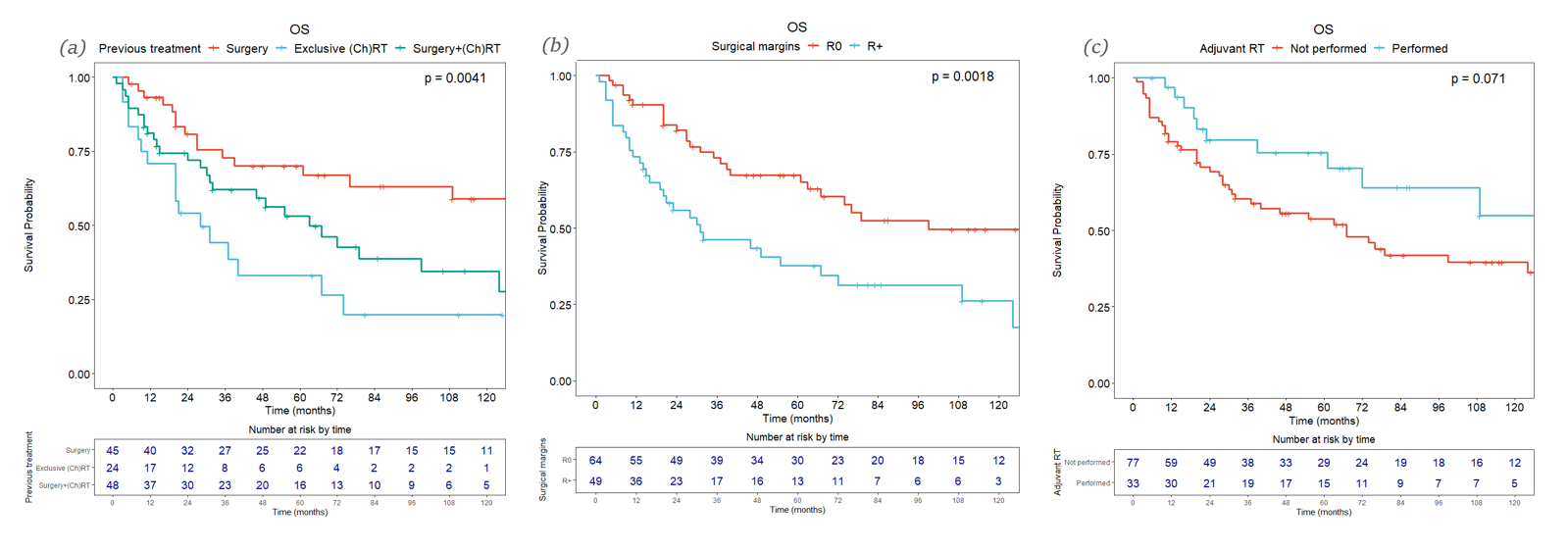
**

**Figure S3.** Kaplan Meier survival curves depicting relapse-free survival (RFS) according to (a) previous treatment (surgery alone, surgery+(Ch)RT, elective (Ch)RT); (b) histologies (“other” include ONB, salivary carcinoma, mesenchymal tumors and SNUC); (c) rpT classification; (d) margin status after salvage surgery for local recurrence.


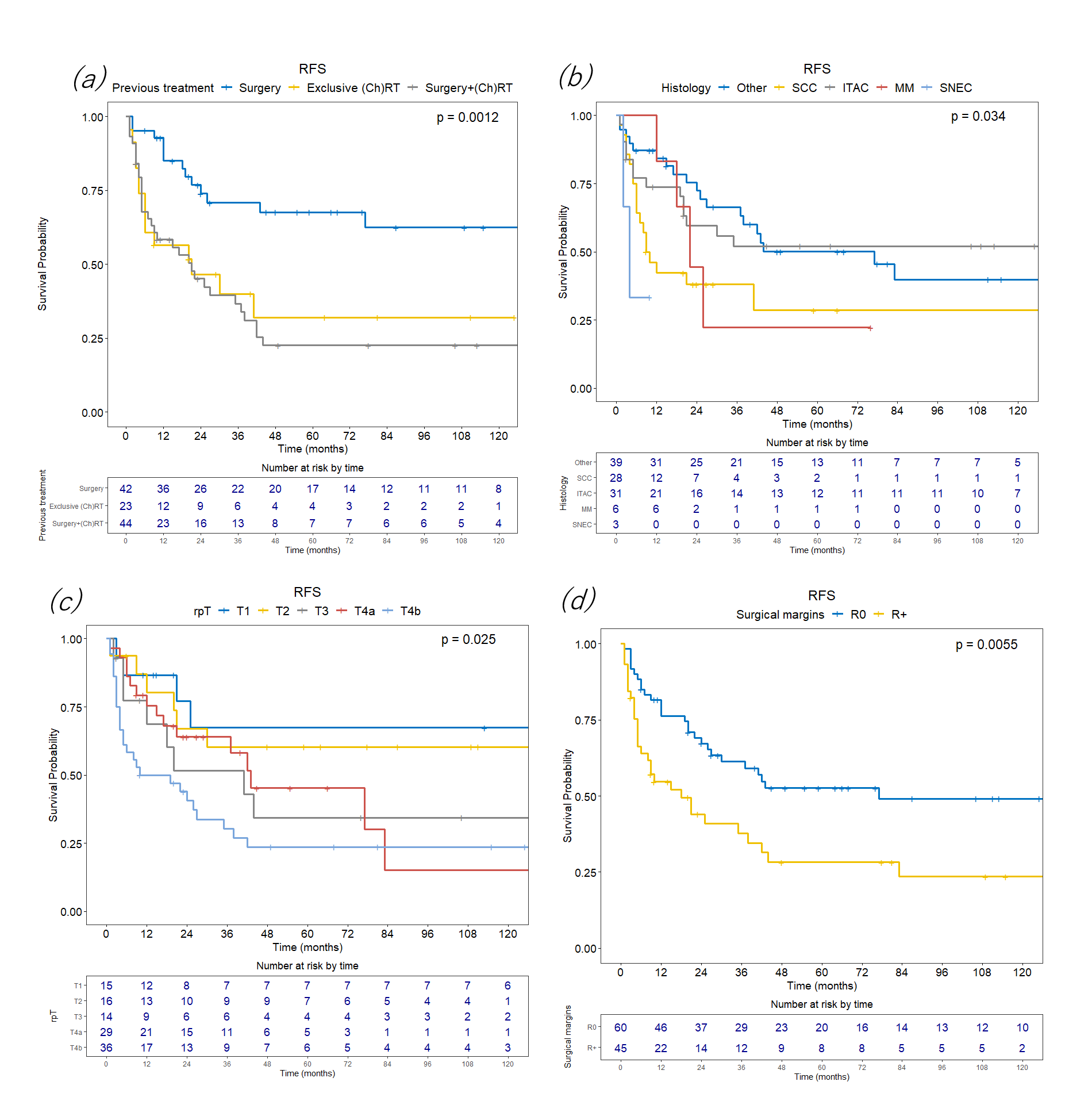

Supplement: Supplementary file 1 — Appendix S1 Supporting Information. [file HED-44-1857-s001.docx]
